# Sex-specific Associations of Aldosterone and Renin With Body Composition: A Population-based Cohort Study

**DOI:** 10.1210/clinem/dgae566

**Published:** 2024-08-16

**Authors:** Gregory L Hundemer, Mohsen Agharazii, François Madore, Marie-Eve Piché, Claudia Gagnon, Alexandra Bussières, Matthieu St-Jean, Alexander A Leung, Gregory A Kline, Manish M Sood, Dylan Burger, Tim Ramsay, Rémi Goupil

**Affiliations:** Department of Medicine, Division of Nephrology, University of Ottawa, Ottawa, ON K1H 7W9, Canada; Ottawa Hospital Research Institute, Ottawa, ON K1Y 4E9, Canada; Department of Medicine, Division of Nephrology, CHU de Québec-Université Laval, Quebec City, QC G1R 3S1, Canada; Department of Medicine, Division of Nephrology, Hôpital du Sacré-Coeur de Montréal, Université de Montréal, Montreal, QC H3C 3J7, Canada; Department of Medicine, Division of Cardiology, Université Laval, Quebec City, QC G1V 0A6, Canada; Institut Universitaire de Cardiologie et de Pneumologie de Québec-Université Laval, Quebec City, QC G1V 4G5, Canada; Department of Medicine, Division of Endocrinology, CHU de Québec-Université Laval, Quebec City, QC G1V 4G2, Canada; Department of Medicine, Division of Endocrinology, University of Sherbrooke, Sherbrooke, QC J1H 5H3, Canada; Department of Medicine, Division of Endocrinology, University of Sherbrooke, Sherbrooke, QC J1H 5H3, Canada; Department of Medicine, Division of Endocrinology and Metabolism, Cumming School of Medicine, University of Calgary, Calgary, AB T2N 1N4, Canada; Department of Community Health Sciences, Cumming School of Medicine, University of Calgary, Calgary, AB T2N 1N4, Canada; Department of Medicine, Division of Endocrinology and Metabolism, Cumming School of Medicine, University of Calgary, Calgary, AB T2N 1N4, Canada; Department of Medicine, Division of Nephrology, University of Ottawa, Ottawa, ON K1H 7W9, Canada; Ottawa Hospital Research Institute, Ottawa, ON K1Y 4E9, Canada; Department of Medicine, Division of Nephrology, University of Ottawa, Ottawa, ON K1H 7W9, Canada; Ottawa Hospital Research Institute, Ottawa, ON K1Y 4E9, Canada; Ottawa Hospital Research Institute, Ottawa, ON K1Y 4E9, Canada; Department of Medicine, Division of Nephrology, Hôpital du Sacré-Coeur de Montréal, Université de Montréal, Montreal, QC H3C 3J7, Canada

**Keywords:** aldosterone, renin, adiposity, body fat, body composition, sex

## Abstract

**Context:**

Renin–angiotensin–aldosterone system (RAAS) activation is closely linked to obesity; however, the sex-specific associations between RAAS activity and body composition among individuals without obesity are not well understood.

**Objective:**

To investigate the associations of aldosterone and renin with body composition according to sex in the general population.

**Design:**

Population-based cohort study.

**Setting:**

Québec (Canada).

**Participants:**

Adults aged 40 to 69 years enrolled in CARTaGENE between 2009 and 2010 (N = 3687).

**Exposures:**

Plasma aldosterone and renin concentrations.

**Main Outcome Measures:**

Body composition assessed via anthropometrics (waist circumference and waist-to-hip ratio), bioelectrical impedance (lean body mass, fat mass, and muscle mass), and cardiac magnetic resonance imaging (epicardial and pericardial adipose tissue volumes).

**Results:**

The mean (SD) age and body mass index were 55 (8) years and 27.3 (4.8) kg/m^2^, respectively. Among males, higher aldosterone and renin were associated with increased waist circumference, increased waist-to-hip ratio, increased fat mass, decreased lean body mass, and decreased muscle mass (*P* < .05). Aldosterone (*P* = .02), but not renin (*P* = .43), was associated with increased ectopic cardiac adiposity in males. In contrast, higher renin (*P* < .05), but not aldosterone (*P* ≥ .05), was associated with increased waist circumference, increased waist-to-hip ratio, and increased cardiac adiposity in females. Among females, higher renin and aldosterone were associated with increased fat mass (*P* < .05) but were not associated with lean body mass or muscle mass (*P* ≥ .05). All aforementioned associations were independent of body weight.

**Conclusion:**

Independent of body weight, increased RAAS activity is associated with unfavorable differences in body composition; however, the strength and pattern of association varies by sex.

When considering an individual's health status and long-term health risks, body composition is more prognostic than body weight (1-4). Although body weight reflects the summative weight of all body tissues, body composition measures the relative proportions of fat and lean mass. Body composition can be assessed in a number of ways, including anthropometric measurements (5-7), bioelectrical impedance analysis (8, 9), and imaging techniques such as dual-energy X-ray absorptiometry (10) and magnetic resonance imaging (MRI) (11). The latter carries the added benefit of measuring ectopic fat, which is linked to increased cardiovascular morbidity and mortality (12).

A bidirectional link between renin-angiotensin-aldosterone system (RAAS) activity and obesity is well-established. Obesity induces RAAS activation, which is considered a primary reason why obesity-related hypertension commonly occurs (13-15). The mechanisms by which obesity causes RAAS activation are complex and include dysregulated adipose tissue secreting angiotensinogen, aldosterone, and mineralocorticoid-releasing factors (13, 14, 16); endothelial dysfunction causing disturbed renal blood flow (14, 16, 17); sympathetic nervous system activation inducing renin secretion (16, 18, 19); and reduced expression of angiotensin-converting enzyme 2 (20-22). On the other hand, RAAS activation may contribute to obesity by increasing food intake via hypothalamic effects (23-25). However, the association between RAAS activity and body composition, particularly among individuals without obesity, is not well understood. Furthermore, because sex influences both body composition and RAAS activity, sex-stratified analyses may yield new insights. Exploration into these sex differences is important as obesity-related hypertension occurs disproportionately in females compared to males (26).

Here, both cross-sectional and prospective analyses were conducted to evaluate the associations of aldosterone and renin with body composition in a large population-based cohort. Body composition was examined in a sex-stratified and weight-independent fashion via assessment of: (1) anthropometric measurements including waist circumference and waist-to-hip ratio, (2) bioelectrical impedance analysis, and (3) cardiac MRI to assess ectopic cardiac adiposity. Participants enrolled in CARTaGENE were studied to test the hypothesis that increased aldosterone and renin concentrations would be associated with unfavorable differences in body composition and to explore variation in these associations by sex.

## Materials and Methods

### Study Design

Cross-sectional and prospective analyses were performed to evaluate sex-specific associations of aldosterone and renin with body composition in a Canadian population-based cohort. All protocols were approved by the Ottawa Health Science Network Research Ethics Board (Protocol ID #20200475-01H). Informed consent was obtained at CARTaGENE study recruitment.

### Data Source and Study Cohort

CARTaGENE (https://cartagene.qc.ca) is an ongoing population-based cohort of 19 996 Québec (Canada) residents designed to study long-term health outcomes (27). Adults aged 40 to 69 years were enrolled between August 2009 and October 2010. Participants were randomly invited using the provincial *Régie de l’assurance maladie du Québec* universal health care administrative database with sampling of 1% of the population base. Participants were selected in such a way that the cohort was representative of the Québec population of this age group on the basis of their sociodemographic characteristics. On enrollment to CARTaGENE, participants were administered a standard questionnaire to assess medical history and medication use. At the enrollment clinic visit, participants had anthropometric measurements captured including height, weight, waist circumference, and hip circumference using standard methods (27, 28). Brachial cuff blood pressure was measured using an Omron HEM-907XL device (Omron Corp., Kyoto, Japan), which measures blood pressure via the oscillometric technique. Resting blood pressure was measured 3 times every 2 minutes, and the mean was reported. Morning upright plasma samples were obtained and were used to measure baseline aldosterone and renin concentrations on a subset of CARTaGENE participants. The baseline characteristics of the subset of participants with aldosterone and renin measurements collected were similar to those of the overall CARTaGENE cohort with the exception of a mild male predominance (27). Participants also underwent bioelectrical impedance analysis to assess body composition at the time of enrollment, as described in the “Outcomes” section. At 5 to 7 years postenrollment, a random subset of CARTaGENE participants was recalled to participate in the Canadian Alliance for Healthy Hearts and Minds (CAHHM) study (29). As part of the CAHHM study, these participants underwent cardiac MRI, which included cardiac adiposity assessment. The only reason for exclusion was unavailable aldosterone or renin measurements from the time of enrollment.

### Exposures: Aldosterone and Renin

Blood samples were obtained at the CARTaGENE enrollment visit with the participant in the seated position, collected on ice, and transferred to the *Biobanque Génome Québec—Centre hospitalier affilié universitaire régional de Chicoutimi* for processing into serum and plasma aliquots for immediate storage at −176 °C. Sampling was performed without any adjustment to participants' usual home medications. Frozen EDTA plasma samples were shipped to the accredited Eastern Ontario Regional Laboratories Association Clinical Biochemistry Laboratory at the Ottawa Hospital General Campus (Ottawa, ON, Canada), where samples were thawed immediately before measurement of aldosterone and renin concentrations. Aldosterone (DiaSorin Cat# 310450, RRID:AB_2889867) and renin (DiaSorin Cat# 310470, RRID:AB_2889866) were measured using chemiluminescent immunoassays on the Liaison analyzer (DiaSorin Inc., Stillwater MN, USA) (30, 31). These assays are used for clinical testing, and regular quality control testing, as part of laboratory quality assurance practices, confirmed imprecision (coefficient of variation) of the aldosterone and renin assays as ≤10% at clinically relevant concentrations. The lower limit of detection for renin was 1.0 ng/L; measures below this were assigned a value of 0.9 ng/L.

### Outcomes

#### Anthropometric measures

Anthropometric measures were captured in CARTaGENE participants at the time of study enrollment. These measures included height and weight (adjusted for in the analyses) along with waist circumference, hip circumference, and waist-to-hip ratio. Waist circumference was measured 3 times using a Seca 200 circumference measuring tape (Seca GmbH & Co., Hamburg, Germany) and following a standard protocol (27, 28). The mean of the 3 waist circumference measurements was calculated, and this value was used in the analyses. Waist circumference and waist-to-hip ratio are measures of central adiposity that provide independent risk information beyond what is captured via body mass index (BMI) (6). Waist circumference and waist-to-hip ratio are strongly linked to heightened cardiometabolic risks including hypertension, insulin resistance, dyslipidemia, coronary artery disease, and mortality (5, 7, 32-36).

#### Bioelectrical impedance analysis

Bioelectrical impedance analysis is a validated method for estimating body composition, where a weak electric current flows through the body and the resistance to flow is measured. From this analysis, body composition measures such as lean body mass, fat mass, water mass, and muscle mass can be determined (37, 38). At CARTaGENE enrollment, participants underwent bioelectrical impedance analysis using the Tanita TBF-310 device (Tanita Corp., Tokyo, Japan) with which foot-to-foot impedance and body weight were captured simultaneously. This device has been validated as an accurate measure of body composition in general healthy adult populations (39, 40).

#### Cardiac MRI measures

The CARTaGENE/CAHHM MRI protocol was previously reported (29). Briefly, participants underwent imaging using a 1.5 Tesla or 3 Tesla magnet. Each center used a test scan for quality assurance that was evaluated and validated by the core laboratory before recruitment. Two-dimensional cine imaging was performed for the entire cardiac cycle in the short-axis plane covering the entire cardiac silhouette by standard steady-state free precession technique. The present study focused on epicardial and pericardial adipose tissue volumes. Although the detrimental effects of abdominal adiposity have long been known, growing contemporary evidence supports ectopic adiposity (eg, myocardial, pericardial, epicardial fat accumulation) playing a major role in cardiovascular pathophysiology including ischemic and nonischemic cardiomyopathy along with accelerated myocardial fibrosis (41-45). Cardiac MRI is highly sensitive to fat and provides accurate, reproducible quantification of cardiac fat accumulation (46-48).

### Statistical Analysis

Baseline characteristics were presented both in the overall cohort as well as stratified by sex. For baseline data, continuous variables were expressed as mean (SD) if normally distributed and as median (25th-75th percentile interquartile range) if nonnormally distributed, whereas categorical variables were expressed as number (%). Aldosterone and renin were log-transformed because of asymmetric distributions. Multivariable linear regression analysis was performed to evaluate associations (based on standardized beta coefficients and 95% CIs) of log-aldosterone and log-renin with the anthropometric, bioelectrical impedance, and cardiac MRI body composition outcomes. These analyses were performed stratifying by sex given the well-established intrinsic differences in body composition between females and males (49). All models were adjusted for the following covariates selected a priori (based on their known influence on aldosterone, renin, and/or body composition) and ascertained at the time of CARTaGENE enrollment: weight, height, age, race/ethnicity, brachial mean arterial blood pressure, serum potassium, serum sodium, estimated glomerular filtration rate, hormone replacement therapy, and antihypertensive medication use by category (ie, separate variables for the following categories: angiotensin-converting enzyme inhibitor, angiotensin II receptor blocker, calcium channel blocker, thiazide diuretic, loop diuretic, potassium-sparing diuretic, and beta blocker). The following additional analyses were performed: (1) stratifying female participants based on menopausal status and (2) further adjusting for 24-hour dietary potassium and sodium intake among participants who completed the Canadian Diet History Questionnaire II (50). All statistical analyses were performed using SAS version 9.4 (SAS Institute, Cary, NC, USA). The 95% CIs that did not overlap with 1.0, and 2-sided *P* values <.05 were considered statistically significant.

## Results

### Baseline Characteristics

**Figure 1.**
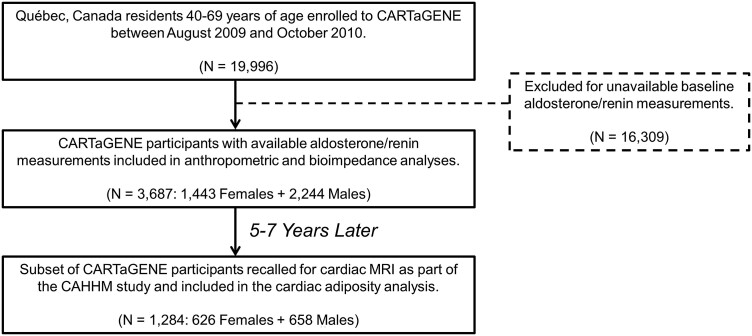
Study flowchart.

Abbreviations: CAHHM, Canadian Alliance for Healthy Hearts and Minds; MRI, magnetic resonance imaging.

From a total of 19 996 CARTaGENE participants, 3687 participants (1443 females, 2244 males) had available aldosterone and renin measurements and underwent anthropometric and bioelectrical impedance measurements and comprised the present study cohort (Fig. 1). Of these, 1284 participants (626 females, 658 males) also underwent cardiac MRI as part of the CAHHM study. The baseline characteristics of the study cohort are displayed in Table 1. The mean (SD) age was 55 (8) years. The majority (91%) of participants self-reported as being White. The mean BMI was 27.3 (4.8) kg/m^2^, with 24% of participants having a BMI ≥ 30 kg/m^2^. The mean systolic and diastolic blood pressure were 130 (14) and 77 (10) mm Hg, respectively. Hypertension (defined as blood pressure ≥140/90 mm Hg and/or use of antihypertensive medication as per International Society of Hypertension thresholds (51)) was present in 27% of participants. Antihypertensive medication use was low among participants (6%). Comorbidities including a history of cardiovascular disease or diabetes mellitus were uncommon at 3% and 7%, respectively. The majority of female participants (63%) were postmenopausal with 16% using hormone replacement therapy. The median (interquartile range) aldosterone and renin concentrations were 218 (161-297) pmol/L and 7.6 (4.6-11.7) ng/L, respectively; the full distributions are displayed elsewhere (see (52)).

**Table 1.** Baseline characteristics of study cohort overall and by sex

Characteristic	Overall cohort	Females	Males
Total (N)	3687	1443	2244
Age, mean (SD)	55 (8)	55 (8)	55 (8)
Race, N (%)			
White	3363 (91)	1352 (94)	2011 (90)
Black	47 (1)	10 (1)	37 (2)
Other	277 (8)	81 (6)	196 (9)
Blood pressure, mm Hg, mean (SD)			
Systolic blood pressure	130 (14)	125 (14)	133 (13)
Diastolic blood pressure	77 (10)	74 (10)	78 (9)
Anthropometrics			
Height, cm, mean (SD)	169 (9)	161 (6)	174 (7)
Weight, kg, mean (SD)	78 (16)	70 (14)	83 (14)
BMI, kg/m^2^, mean (SD)	27.3 (4.8)	26.9 (5.4)	27.6 (4.3)
Obesity (BMI ≥30 kg/m^2^), N (%)	887 (24)	337 (23)	550 (25)
Comorbidities, N (%)			
Cardiovascular disease	117 (3)	36 (2)	81 (4)
Hypertension*^a^*	991 (27)	307 (21)	684 (30)
Diabetes mellitus	270 (7)	80 (6)	190 (8)
Active smoking	787 (21)	263 (18)	524 (23)
Postmenopausal, N (%)	—	903 (63)	—
Daily dietary intake, mmol/day, mean (SD)*^b^*			
Sodium	108 (68)	100 (59)	115 (74)
Potassium	88 (47)	86 (46)	89 (49)
Laboratory measurements			
Aldosterone, pmol/L, Median (IQR)	218 (161-297)	225 (166-315)	213 (158-287)
Renin, ng/L, Median (IQR)	7.6 (4.6-11.7)	6.3 (3.5-9.9)	8.4 (5.4-12.7)
eGFR, mL/min/1.73 m^2^, Mean (SD)	87 (14)	87 (14)	88 (13)
Sodium, mmol/L, mean (SD)	139 (2)	139 (2)	139 (2)
Potassium, mmol/L, mean (SD)	4.3 (0.5)	4.3 (0.4)	4.4 (0.5)
Albumin, g/L, mean (SD)	43 (3)	42 (3)	43 (3)
Glucose, mmol/L, mean (SD)	5.8 (1.6)	5.5 (1.4)	5.9 (1.8)
Hemoglobin A1c, %, Mean (SD)	5.7 (0.6)	5.7 (0.5)	5.7 (0.7)
Total cholesterol, mmol/L, mean (SD)	5.24 (1.01)	5.38 (0.98)	5.16 (1.01)
HDL cholesterol, mmol/L, mean (SD)	1.22 (0.41)	1.42 (0.42)	1.09 (0.34)
LDL cholesterol, mmol/L, mean (SD)	3.17 (0.90)	3.22 (0.88)	3.14 (0.90)
TSH, mU/L, mean (SD)	1.92 (1.39)	1.92 (1.43)	1.93 (1.35)
Thyroxine, pmol/L, mean (SD)	11.3 (2.5)	11.3 (2.6)	11.4 (2.4)
Antihypertensive medication use, N (%)			
Any antihypertensive medication	213 (6)	97 (7)	116 (5)
ACE inhibitor	55 (1)	20 (1)	35 (2)
ARB	110 (3)	51 (4)	59 (2)
Calcium channel blocker	51 (1)	18 (1)	33 (1)
Thiazide diuretic	66 (2)	35 (2)	31 (1)
Loop diuretic	3 (0)	1 (0)	2 (0)
Potassium-sparing diuretic	12 (0)	7 (0)	5 (0)
Beta blocker	38 (1)	22 (1)	16 (1)
Other medication use, N (%)			
Aspirin	444 (12)	125 (9)	319 (14)
Statin	609 (17)	190 (13)	419 (19)
Metformin	129 (4)	44 (3)	85 (4)
Hormone replacement therapy	—	230 (16)	—
Cardiac MRI performed			
N (%)	1284 (35)	626 (43)	658 (29)
Time from study entry to MRI, y, mean (SD)	6.2 (0.5)	6.2 (0.5)	6.2 (0.5)

Abbreviations: ACE, angiotensin-converting enzyme; ARB, angiotensin II receptor blocker; BMI, body mass index; eGFR, estimated glomerular filtration rate; HDL, high-density lipoprotein; IQR, interquartile range; LDL, low-density lipoprotein.

^*a*^Hypertension defined as blood pressure ≥140/90 mmHg and/or use of antihypertensive medication.

^*b*^Assessed via the Canadian Diet History Questionnaire II (50). Data available for 2055 participants (56%) in the overall cohort.

### Anthropometric Measures

**Figure 2.**
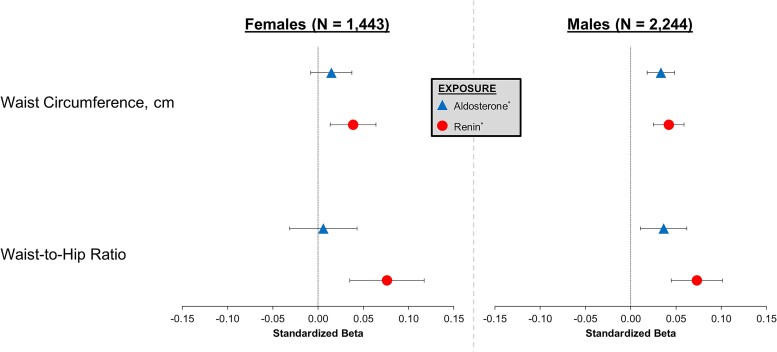
Sex-specific associations of aldosterone and renin with baseline anthropometric body composition measurements (N = 3687). Anthropometric body composition measurements performed at CARTaGENE study entry (ie, at the time of aldosterone and renin measurements). Models adjusted for weight, height, age, race, brachial mean arterial blood pressure, serum potassium, serum sodium, estimated glomerular filtration rate, hormone replacement therapy, and antihypertensive medication use. *Log-transformed variable.

The mean (SD) waist circumference in females and males were 88 (13) cm and 98 (12) cm, respectively. The mean (SD) waist-to-hip ratio in females and males were 0.86 (0.07) and 0.98 (0.07), respectively. Figure 2 displays the cross-sectional, sex-stratified, and multivariable-adjusted associations of log-aldosterone and log-renin with waist circumference and waist-to-hip ratio. Among females, log-aldosterone was not significantly associated with either waist circumference (β = 0.015, *P* = .21) or waist-to-hip ratio (β = 0.006, *P* = .76). However, log-renin was associated with both greater waist circumference (β = 0.039, *P* = .003) and greater waist-to-hip ratio (β = 0.076, *P* < .001). Among males, log-aldosterone was associated with greater waist circumference (β = 0.033, *P* < .001) and greater waist-to-hip ratio (β = 0.036, *P* = .005). Similarly, log-renin was also associated with greater waist circumference (β = 0.042, *P* < .001) and greater waist-to-hip ratio (β = 0.073, *P* < .001).

### Bioelectrical Impedance Analysis

**Figure 3.**
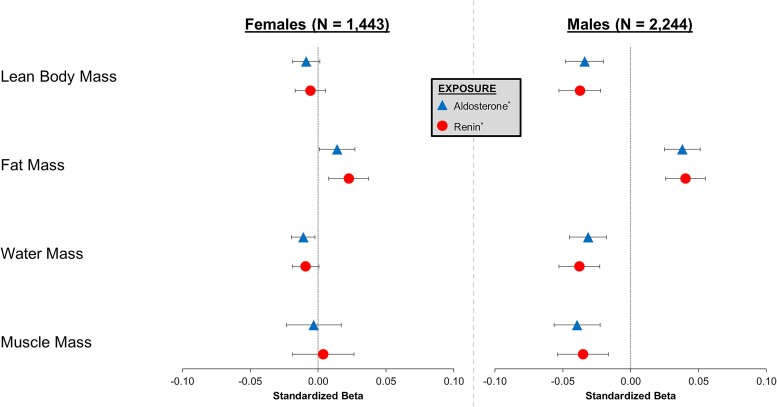
Sex-specific associations of aldosterone and renin with baseline bioelectrical impedance body composition measurements (N = 3687). Bioelectrical body composition measurements performed at CARTaGENE study entry (ie, at the time of aldosterone and renin measurements). Models adjusted for weight, height, age, race, brachial mean arterial blood pressure, serum potassium, serum sodium, estimated glomerular filtration rate, hormone replacement therapy, and antihypertensive medication use. *Log-transformed variable.

Figure 3 displays the cross-sectional, sex-stratified, and multivariable-adjusted associations of log-aldosterone and log-renin with lean body mass, fat mass, water mass, and muscle mass. Among females, log-aldosterone was associated with increased fat mass (β = 0.014, *P* = .04) and decreased water mass (β = −0.011, *P* = .01). However, there was no significant association between log-aldosterone and either lean body mass (β = −0.009, *P* = .08) or muscle mass (β = −0.003, *P* = .75). Log-renin was significantly associated with increased fat mass (β = 0.023, *P* = .003) but not with lean body mass (β = −0.006, *P* = .33), water mass (β = −0.009, *P* = .06), or muscle mass (β = 0.004, *P* = .75). Among males, log-aldosterone was associated with decreased lean body mass (β = −0.034, *P* < .001), increased fat mass (β = 0.038, *P* < .001), decreased water mass (β = −0.031, *P* < .001), and decreased muscle mass (β = −0.040, *P* < .001). Similarly, log-renin was also associated with decreased lean body mass (β = −0.037, *P* < .001), increased fat mass (β = 0.040, *P* < .001), decreased water mass (β = −0.038, *P* < .001), and decreased muscle mass (β = −0.035, *P* < .001).

### Cardiac MRI Measures

**Figure 4.**
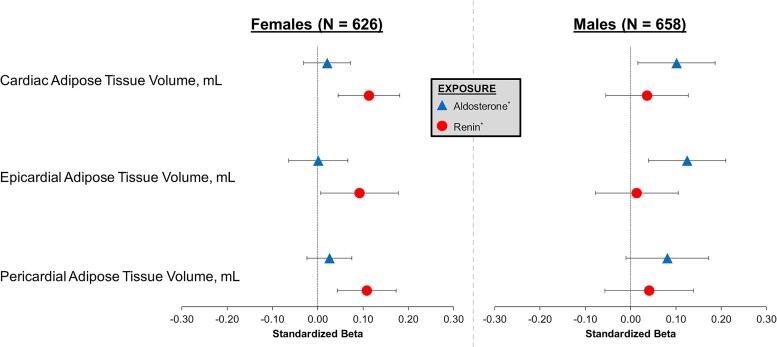
Sex-specific associations of aldosterone and renin with cardiac MRI adiposity measurements at the 5 to 7 year follow-up visit (n = 1284). Outcomes based on cardiac MRI 5 to 7 years after CARTaGENE enrollment (ie, 5-7 years after initial aldosterone and renin measurements). Models adjusted for weight, height, age, race, brachial mean arterial blood pressure, serum potassium, serum sodium, estimated glomerular filtration rate, hormone replacement therapy, and antihypertensive medication use. *Log-transformed variable.

Abbreviation: MRI, magnetic resonance imaging.

Among the present study cohort, 1284 participants underwent cardiac MRI, including cardiac adiposity assessment. Figure 4 displays the prospective, sex-stratified, and multivariable-adjusted associations of log-aldosterone and log-renin with cardiac, epicardial, and pericardial adipose tissue volumes. Among females, there was no statistical association between log-aldosterone and either cardiac (β = 0.021, *P* = .43), epicardial (β = 0.001, *P* = .97), or pericardial (β = 0.026, *P* = .31) adipose tissue volumes. However, log-renin was associated with greater cardiac (β = 0.113, *P* = .001), epicardial (β = 0.092, *P* = .04), and pericardial (β = 0.108, *P* = .001) adipose tissue volumes. Among males, log-aldosterone was associated with greater cardiac (β = 0.102, *P* = .02) and epicardial (β = 0.125, *P* = .004) adipose tissue volumes. However, log-aldosterone was not significantly associated with pericardial adipose tissue volume (β = 0.081, *P* = .08). Log-renin was not significantly associated with either cardiac (β = 0.036, *P* = .43), epicardial (β = 0.013, *P* = .77), or pericardial (β = 0.041, *P* = .41) adipose tissue volumes.

### Additional Analyses

Additional analyses comparing premenopausal (n = 540) and postmenopausal (n = 903) females are displayed elsewhere (see (52)). Similar overall trends were seen for both the anthropometric and bioelectrical impedance outcomes (52). For the cardiac MRI outcomes, log-renin associations were similar between premenopausal and postmenopausal females; however, log-aldosterone was associated with greater cardiac and pericardial adipose tissue volumes in premenopausal females but not in postmenopausal females (52). Separate analyses adjusting for 24-hour dietary potassium and sodium intake among participants who completed the Canadian Diet History Questionnaire II (available in 2055/3687 [56%] of the overall cohort) yielded similar results to the primary analyses (52).

## Discussion

This large population-based cohort study of Canadian adults aged 40 to 69 years measured the sex-specific associations of aldosterone and renin concentrations with body composition. Although increased RAAS activity was generally associated with unfavorable differences in body composition, the strength and pattern of these associations varied between females and males. Among males, higher aldosterone and renin were associated with increased waist circumference and waist-to-hip ratio, whereas only renin was significantly associated with these anthropometric measures in females. Furthermore, among males, higher aldosterone and renin were associated with increased fat mass along with decreased lean body mass and muscle mass. In contrast, among females, higher aldosterone and renin were significantly associated only with fat mass but not lean body mass or muscle mass. Contrasting associations by sex were found in regard to ectopic cardiac adiposity with higher aldosterone (but not renin) being associated among males while higher renin (but not aldosterone) being associated among females. Notably, CARTaGENE is population-based cohort which consisted predominantly of individuals with BMI <30 kg/m^2^. All analyses controlled for baseline weight, thereby suggesting that these associations between aldosterone, renin, and body composition were independent of body weight.

These findings confirm and extend the results of prior work. The bulk of the existing literature on RAAS activation and body composition focuses specifically on individuals living with obesity (13-15). These studies consistently show increased RAAS activation among individuals with BMI ≥30 kg/m^2^, which contributes to the exceedingly high correlation between obesity and hypertension (53, 54). In fact, the prevalence of hypertension among individuals living with obesity is approximately double that of individuals living without obesity, and the severity of obesity parallels the prevalence of hypertension (55). The present study cohort predominantly consisted of individuals with a BMI <30 kg/m^2^. We now show that increased RAAS activity associates with increased central adiposity, increased fat mass, decreased lean body mass, and increased ectopic cardiac adiposity even among a population with a BMI mostly <30 kg/m^2^ and independent of body weight. Importantly, each of these unfavorable differences in body composition is associated with increased cardiovascular morbidity and mortality risk beyond what is captured via traditional measures such as body weight or BMI (5-7, 12, 32-36, 42, 43, 45, 56).

One important finding from the present study was that the associations of aldosterone and renin with body composition were generally in the same direction, even if nonstatistically significant. This suggests renin-dependent (ie, “secondary”) aldosteronism rather than renin-independent (ie, “primary”) aldosteronism. This distinction is important given the well-established link between aldosterone and obesity with these 2 patterns of aldosterone secretion representing varying pathophysiologic pathways (renin/angiotensin II-mediated aldosterone secretion vs autonomous aldosterone secretion), which both associate with obesity (57, 58). Although the majority of the existing literature supports predominantly renin-dependent aldosteronism in obesity, evidence among patients with primary aldosteronism also supports a link of renin-independent aldosteronism to obesity (59, 60). In the present cohort, where most participants did not exhibit hypertension or obesity, we found the association to be predominantly of a renin-dependent nature.

This study is also novel in exploring sex-specific associations between aldosterone and renin concentrations with body composition. Although a link between increased RAAS activity and unfavorable differences in body composition was found in both females and males, the associations were more pronounced in males than in females (both premenopausal and postmenopausal). Further, sex differences in the patterns of association between RAAS activity and cardiac adiposity were noted. Specifically, renin (but not aldosterone) was associated with cardiac adiposity in females, whereas aldosterone (but not renin) was associated with cardiac adiposity in males. Although our study was not designed to determine the underlying etiologies that explain these variations, they may relate to intrinsic differences in RAAS activation between sexes. Males generally have more visceral adipose tissue, which fails the hyperplastic process and mostly undergoes hypertrophy and may therefore be more subject to the effects of dysfunctional adipose tissue (eg, increased secretion of aldosterone and other secretagogues locally) (61). In contrast, females have more subcutaneous white adipose tissue capable of hyperplasia and higher circulating levels of leptin, which stimulates renin secretion (16, 19, 61, 62). Additionally, there are inherent sex-related differences in aldosterone biosynthesis pathways because of factors such as steroidogenic acute regulatory protein and CYP11A1 (63). Perhaps there may also be an estrogen-related component, yet to be clearly defined, given some of the differences found between premenopausal vs postmenopausal females within the present study. Future studies will be necessary to fully delineate not only the biological mechanisms underlying these observed sex differences but also the long-term clinical implications.

The strengths and novelty of this study include a large population-based cohort in which data collection was performed using standardized methods thus limiting ascertainment bias. Furthermore, multiple different measures of body composition were measured, both cross-sectionally and also prospectively, and produced consistent findings across all modalities. Finally, the standardized incorporation of cardiac MRI into the study protocol is novel and provided what is considered the gold standard for assessing ectopic cardiac fat deposition.

Our work must be interpreted within the context of several limitations. First, this study is observational; therefore, we were able to identify association but not causation. We did adjust for important variables known to influence aldosterone, renin, and body composition within our multivariable models; however, unmeasured confounding may still have been present. Second, the study population consisted exclusively of adult residents from Québec, Canada. Thus, the results may not be generalizable to other populations. Specifically, the population of Québec is predominantly of White race. Given known differences in RAAS activity among predominantly Black populations, this may particularly limit generalizability to Black individuals (64). Third, although the subset of CARTaGENE participants that had available aldosterone and renin measurements (and were thus included in this study) had overall similar baseline characteristics to those of the overall CARTaGENE cohort (27), there was a male predominance (61%) compared to the overall CARTaGENE cohort. This may have limited the statistical power to identify associations between RAAS activity and body composition in females relative to males. Fourth, bioelectrical impedance analysis may underestimate body fat (8). Any resultant bias from this potential underestimation would be anticipated to affect all participants equally. Moreover, our results were consistent across other body composition modalities. Fifth, aldosterone and renin were measured on an ad libitum diet with regard to sodium and potassium intake. However, a sensitivity analysis including only participants who had information collected on daily sodium and potassium intake yielded similar results to the primary analysis. Sixth, cardiac MRI was performed 5 to 7 years following CARTaGENE entry (ie, the time of aldosterone/renin, anthropometric, and bioelectrical impedance measurements), which may have allowed sufficient time for changes in body composition. However, similar associations were seen with the cardiac MRI adiposity measures as with the body composition measures captured at study entry. Seventh, single measurements of aldosterone and renin have been shown to display significant intraindividual variability, which may have resulted in some degree of misclassification (65). Finally, it is well established that adipose tissue possesses a local RAAS, including aldosterone synthase and renin (66-69). However, as aldosterone and renin were only measured in plasma samples, it is not possible to know whether intra-adipose RAAS is playing a role in the relationships observed in the present study.

In summary, this novel population-based study demonstrated that independent of body weight, increased aldosterone and renin concentrations were associated with unfavorable differences in body composition. These differences included increased central adiposity, fat mass, and ectopic cardiac adiposity. The strength and patterns of these associations varied by sex. Future studies are needed to enhance our understanding of the biochemical mechanisms underlying these findings.

## Data Availability

Some or all datasets generated during and/or analyzed during the current study are not publicly available but are available from the corresponding author on reasonable request.

## Abbreviations

BMI: body mass index
CAHHM: Canadian Alliance for Healthy Hearts and Minds
MRI: magnetic resonance imaging
RAAS: renin–angiotensin–aldosterone system
